# Supplementary material for: Spatial heterogeneity of coral reef benthic communities in Kenya
Source: PLoS One. 2020 Aug 26;15(8):e0237397. doi: 10.1371/journal.pone.0237397 (PMC7449394; doi:10.1371/journal.pone.0237397)
Supplement: S1 Table — A table of sites sampled according to their geographical zones, habitat factors and management levels. (DOCX) [file pone.0237397.s001.docx]

| **Geographic zone** | **No. of reefs**  **(zone total)** | **Habitat factors & management status** | **No. of reefs per level in each factor** |
| --- | --- | --- | --- |
| North | 20 | Exposure | Exposed 12; Sheltered 8 |
|  |  | Depth | Deep 5; Shallow 15 |
|  |  | Reef type | Channel 3; Lagoon 6; Fringing 5; Patch 6 |
|  |  | Management | Park 0; Reserve 17; Unprotected 3 |
| Central | 10 | Exposure | Exposed 6; Sheltered 4 |
|  |  | Depth | Deep 5; Shallow 5 |
|  |  | Reef type | Channel 0; Lagoon 2; Fringing 5; Patch 3 |
|  |  | Management | Park 6; Reserve 0; Unprotected 4 |
| South | 8 | Exposure | Exposed 3; Sheltered 5 |
|  |  | Depth | Deep 4; Shallow 4 |
|  |  | Reef type | Channel 2; Lagoon 0; Fringing 5; Patch 1 |
|  |  | Management | Park 4; Reserve 2; Unprotected 2 |
