# Supplementary material for: Spatial heterogeneity of coral reef benthic communities in Kenya
Source: PLoS One. 2020 Aug 26;15(8):e0237397. doi: 10.1371/journal.pone.0237397 (PMC7449394; doi:10.1371/journal.pone.0237397)
Supplement: S2 Table — Percentage cover of major benthic categories recorded at all surveyed coral reef sites along the Kenyan coast. (DOCX) [file pone.0237397.s002.docx]

| Site name | Geographical zone | Depth category | Depth-m | Exposure | Reef type | Management | Percentage benthic cover per site (selected variables only) | | | | | | | | | |
| --- | --- | --- | --- | --- | --- | --- | --- | --- | --- | --- | --- | --- | --- | --- | --- | --- |
|  |  |  |  |  |  |  | Coralline algae | Halimeda | Macroalgae | Turf algae | Hard coral | Seagrass | Soft corals | Dead standing coral | Other-Invertebrates | Recently dead coral |
| Anthias | Central | Deep | 16 | Exposed | Fringing | Unprotected | 0.00 | 9.00 | 12.00 | 29.33 | 18.00 | 0.00 | 22.00 | 1.00 | 0.67 | 0.67 |
| Boso | North | Shallow | 4 | Sheltered | Lagoon | Reserve | 3.20 | 12.32 | 3.68 | 37.28 | 14.88 | 14.56 | 3.52 | 0.00 | 1.12 | 0.00 |
| Chole | North | Shallow | 2 | Exposed | Lagoon | Reserve | 12.96 | 0.00 | 1.12 | 18.24 | 36.00 | 0.00 | 0.00 | 0.96 | 0.96 | 0.00 |
| Chongo cha Bomani | North | Deep | 18 | Exposed | Patch | Reserve | 0.96 | 6.44 | 7.88 | 47.88 | 16.60 | 0.00 | 6.48 | 0.00 | 5.56 | 0.00 |
| Chongo cha Chano | North | Deep | 18 | Exposed | Patch | Reserve | 42.56 | 0.64 | 0.00 | 2.40 | 4.32 | 0.00 | 9.12 | 0.00 | 9.28 | 0.00 |
| Chongo cha Mvundeni | North | Deep | 15 | Exposed | Patch | Reserve | 25.92 | 17.76 | 1.12 | 3.04 | 6.08 | 0.00 | 6.08 | 0.00 | 3.04 | 0.00 |
| Chongo cha Mwongo Shariff | North | Deep | 18 | Exposed | Patch | Reserve | 27.83 | 24.70 | 0.35 | 1.22 | 3.65 | 0.00 | 7.48 | 0.00 | 0.35 | 0.00 |
| Chongo cha Rubu | North | Deep | 18 | Exposed | Patch | Reserve | 21.12 | 16.16 | 3.52 | 12.80 | 7.52 | 0.00 | 1.44 | 0.00 | 0.80 | 0.00 |
| Dolphin | Central | Deep | 13 | Exposed | Fringing | Unprotected | 4.33 | 6.33 | 1.00 | 46.33 | 6.33 | 0.00 | 23.33 | 0.33 | 0.33 | 0.67 |
| Drummers | Central | Deep | 13 | Exposed | Fringing | Unprotected | 3.00 | 10.33 | 1.67 | 49.33 | 8.00 | 0.00 | 11.67 | 0.00 | 0.67 | 0.00 |
| Fawacho | North | Shallow | 5 | Sheltered | Channel | Unprotected | 0.17 | 0.33 | 30.33 | 3.33 | 42.67 | 0.00 | 0.00 | 0.33 | 2.17 | 0.00 |
| Kibuyuni | South | Shallow | 2 | Sheltered | Channel | Unprotected | 1.50 | 0.00 | 2.67 | 7.00 | 52.33 | 2.67 | 11.67 | 2.33 | 0.50 | 0.67 |
| Kishanga | North | Shallow | 3 | Sheltered | Lagoon | Reserve | 14.16 | 12.76 | 8.56 | 24.36 | 21.12 | 0.00 | 13.12 | 0.00 | 0.32 | 0.00 |
| Kisite Deep | South | Deep | 10 | Sheltered | Fringing | Park | 0.00 | 0.00 | 0.00 | 0.00 | 15.00 | 0.00 | 41.00 | 3.67 | 0.33 | 1.00 |
| Kisite Leeward | South | Shallow | 5 | Sheltered | Fringing | Park | 1.00 | 0.00 | 1.00 | 0.00 | 36.33 | 0.00 | 6.67 | 0.67 | 0.33 | 0.00 |
| Kisite Seaward | South | Shallow | 6 | Exposed | Fringing | Park | 0.25 | 0.00 | 0.00 | 2.00 | 35.50 | 0.00 | 33.25 | 0.50 | 0.25 | 1.00 |
| Kui | North | Shallow | 5 | Sheltered | Lagoon | Reserve | 8.17 | 25.67 | 4.17 | 5.67 | 12.50 | 0.00 | 9.17 | 1.00 | 1.50 | 1.33 |
| Kupi | North | Shallow | 4 | Sheltered | Lagoon | Reserve | 0.17 | 0.00 | 3.50 | 26.00 | 40.33 | 0.00 | 0.17 | 0.33 | 0.83 | 0.17 |
| Kwa Radi | North | Shallow | 2 | Exposed | Fringing | Reserve | 8.64 | 14.28 | 17.36 | 33.04 | 17.48 | 2.88 | 1.28 | 0.00 | 1.76 | 0.00 |
| Mabiyu | North | Shallow | 5 | Exposed | Channel | Unprotected | 1.33 | 1.00 | 11.33 | 2.17 | 29.00 | 0.33 | 1.50 | 1.33 | 4.17 | 0.00 |
| Makokokwe | South | Deep | 11 | Exposed | Patch | Park | 2.75 | 0.50 | 9.50 | 3.25 | 47.00 | 0.00 | 11.50 | 3.75 | 0.00 | 1.75 |
| Mikes Inner | North | Shallow | 3 | Sheltered | Channel | Reserve | 0.32 | 2.56 | 1.28 | 30.08 | 50.40 | 0.00 | 0.00 | 0.00 | 1.28 | 0.16 |
| Mikes Outer | North | Shallow | 3 | Exposed | Patch | Reserve | 0.80 | 9.44 | 36.48 | 21.28 | 15.04 | 1.28 | 0.80 | 0.00 | 0.64 | 0.00 |
| Mkokoni | North | Shallow | 3 | Sheltered | Lagoon | Reserve | 0.96 | 7.20 | 17.76 | 38.56 | 33.12 | 1.60 | 0.16 | 0.00 | 0.00 | 0.00 |
| Mkwiro | South | Shallow | 4 | Sheltered | Channel | Unprotected | 2.17 | 0.17 | 56.83 | 5.50 | 20.67 | 0.00 | 2.50 | 1.33 | 4.00 | 0.33 |
| Mlango wa Muhindi | North | Shallow | 4.5 | Exposed | Fringing | Reserve | 0.16 | 17.60 | 0.48 | 25.64 | 43.48 | 2.32 | 3.20 | 0.00 | 1.16 | 0.00 |
| Moray | Central | Deep | 12 | Exposed | Fringing | Unprotected | 0.33 | 1.33 | 3.67 | 38.33 | 6.67 | 0.00 | 41.33 | 0.33 | 0.67 | 0.00 |
| New Coral Gardens | Central | Shallow | 1 | Sheltered | Patch | Park | 1.17 | 0.33 | 1.83 | 2.33 | 49.33 | 0.00 | 0.67 | 2.83 | 0.67 | 0.67 |
| North Reef | Central | Shallow | 3 | Exposed | Patch | Park | 0.17 | 8.67 | 6.17 | 9.17 | 16.33 | 0.17 | 1.67 | 0.50 | 1.00 | 0.00 |
| Old Coral Gardens | Central | Shallow | 1 | Sheltered | Patch | Park | 8.33 | 0.00 | 5.17 | 2.33 | 29.50 | 0.00 | 2.33 | 0.17 | 0.33 | 0.00 |
| Pezzali | North | Shallow | 6 | Exposed | Fringing | Unprotected | 4.00 | 0.33 | 47.17 | 5.67 | 11.67 | 0.00 | 0.00 | 0.17 | 2.00 | 0.00 |
| Richard Bennette | Central | Shallow | 1.8 | Sheltered | Lagoon | Park | 6.33 | 10.00 | 26.00 | 19.33 | 29.00 | 0.00 | 0.00 | 0.00 | 0.00 | 0.00 |
| Shili | North | Shallow | 2 | Sheltered | Fringing | Reserve | 3.52 | 1.44 | 41.60 | 10.72 | 10.72 | 6.56 | 0.64 | 0.32 | 1.28 | 0.00 |
| Shimo La Tewa | North | Shallow | 5 | Exposed | Fringing | Reserve | 1.83 | 2.67 | 27.50 | 3.50 | 33.33 | 0.67 | 10.33 | 0.00 | 2.00 | 0.33 |
| Turtle Reef | Central | Deep | 7 | Exposed | Fringing | Park | 8.67 | 10.67 | 47.67 | 9.33 | 8.67 | 0.67 | 1.33 | 0.67 | 0.33 | 1.00 |
| Upper Mpunguti Leeward | South | Deep | 6.5 | Sheltered | Fringing | Reserve | 1.33 | 0.00 | 11.67 | 15.00 | 18.00 | 0.00 | 17.67 | 0.00 | 4.00 | 0.67 |
| Upper Mpunguti Seaward | South | Deep | 7 | Exposed | Fringing | Reserve | 11.33 | 0.00 | 2.67 | 20.00 | 16.67 | 0.00 | 37.67 | 0.00 | 0.67 | 0.67 |
| Watamu Coral Garden | Central | Shallow | 1.8 | Sheltered | Lagoon | Park | 2.33 | 3.67 | 25.67 | 30.33 | 28.00 | 0.00 | 0.67 | 0.33 | 0.00 | 0.00 |
