# Supplementary material for: Spatial heterogeneity of coral reef benthic communities in Kenya
Source: PLoS One. 2020 Aug 26;15(8):e0237397. doi: 10.1371/journal.pone.0237397 (PMC7449394; doi:10.1371/journal.pone.0237397)
Supplement: S3 Table — Percentage cover of coral genera recorded at all surveyed coral reef sites along the Kenyan coast. (DOCX) [file pone.0237397.s003.docx]

| Geographical zone | Depth category | Depth-m | Exposure | Reef type | Management | Percentage cover of coral genera | | | | | | | | | | | | |
| --- | --- | --- | --- | --- | --- | --- | --- | --- | --- | --- | --- | --- | --- | --- | --- | --- | --- | --- |
|  |  |  |  |  |  | Acropora | Echinopora | Favites | Galaxea | Gardineroseris | Goniastrea | Goniopora | Leptoria | Lobophyllia | Montipora | Platygyra | Pocillopora | Porites |
| Central | Deep | 16 | Exposed | Fringing | Unprotected | 4.00 | 0.00 | 1.00 | 0.33 | 0.00 | 0.00 | 0.00 | 0.00 | 6.67 | 3.00 | 0.00 | 0.00 | 0.00 |
| North | Shallow | 4 | Sheltered | Lagoon | Reserve | 5.44 | 0.64 | 0.16 | 0.00 | 0.48 | 0.00 | 0.00 | 0.00 | 0.00 | 0.00 | 0.00 | 1.44 | 6.72 |
| North | Shallow | 2 | Exposed | Lagoon | Reserve | 0.00 | 0.00 | 3.20 | 0.32 | 0.80 | 0.00 | 0.00 | 2.88 | 0.00 | 5.92 | 0.00 | 0.00 | 16.32 |
| North | Deep | 18 | Exposed | Patch | Reserve | 0.00 | 6.24 | 2.72 | 0.00 | 0.00 | 0.00 | 0.00 | 0.00 | 1.76 | 0.00 | 0.96 | 0.00 | 2.20 |
| North | Deep | 18 | Exposed | Patch | Reserve | 0.00 | 0.00 | 0.00 | 0.00 | 0.00 | 0.00 | 0.00 | 0.00 | 0.00 | 2.24 | 0.32 | 0.00 | 1.60 |
| North | Deep | 15 | Exposed | Patch | Reserve | 0.00 | 0.00 | 1.12 | 0.64 | 0.00 | 0.00 | 0.00 | 0.00 | 0.00 | 2.88 | 0.16 | 0.16 | 0.48 |
| North | Deep | 18 | Exposed | Patch | Reserve | 0.50 | 0.00 | 1.00 | 0.00 | 0.00 | 0.00 | 0.00 | 0.00 | 0.00 | 1.17 | 0.00 | 0.33 | 1.00 |
| North | Deep | 18 | Exposed | Patch | Reserve | 2.08 | 0.00 | 0.16 | 0.00 | 0.00 | 0.00 | 0.00 | 0.00 | 0.00 | 3.36 | 0.16 | 0.16 | 0.32 |
| Central | Deep | 13 | Exposed | Fringing | Unprotected | 3.00 | 0.00 | 0.00 | 0.00 | 0.00 | 0.00 | 0.00 | 0.00 | 0.00 | 0.33 | 0.00 | 0.33 | 2.67 |
| Central | Deep | 13 | Exposed | Fringing | Unprotected | 5.67 | 0.00 | 0.33 | 0.00 | 0.00 | 0.00 | 0.00 | 0.00 | 0.00 | 0.00 | 1.33 | 0.33 | 0.33 |
| North | Shallow | 5 | Sheltered | Channel | Unprotected | 0.00 | 0.83 | 1.83 | 11.00 | 0.00 | 0.00 | 0.00 | 0.00 | 0.00 | 0.50 | 2.00 | 0.17 | 16.67 |
| South | Shallow | 2 | Sheltered | Channel | Unprotected | 0.00 | 7.83 | 1.83 | 1.83 | 0.00 | 0.00 | 1.33 | 0.00 | 6.50 | 0.33 | 1.67 | 0.00 | 18.67 |
| North | Shallow | 3 | Sheltered | Lagoon | Reserve | 0.64 | 1.12 | 1.12 | 0.00 | 6.72 | 0.00 | 0.00 | 1.12 | 0.00 | 0.00 | 0.64 | 0.80 | 8.80 |
| South | Deep | 10 | Sheltered | Fringing | Park | 9.33 | 0.67 | 1.67 | 0.00 | 0.00 | 0.00 | 0.00 | 0.33 | 0.00 | 0.00 | 0.00 | 0.00 | 3.00 |
| South | Shallow | 5 | Sheltered | Fringing | Park | 3.67 | 7.00 | 0.00 | 0.33 | 0.00 | 0.00 | 21.33 | 0.00 | 0.00 | 0.00 | 0.00 | 0.00 | 2.00 |
| South | Shallow | 6 | Exposed | Fringing | Park | 11.75 | 0.25 | 0.25 | 0.00 | 0.00 | 0.00 | 14.00 | 0.00 | 0.00 | 0.00 | 0.00 | 0.00 | 9.00 |
| North | Shallow | 5 | Sheltered | Lagoon | Reserve | 3.83 | 0.00 | 0.50 | 0.33 | 0.00 | 0.00 | 0.00 | 0.00 | 0.00 | 0.00 | 0.00 | 0.33 | 6.00 |
| North | Shallow | 4 | Sheltered | Lagoon | Reserve | 2.67 | 2.83 | 3.50 | 0.00 | 0.00 | 0.00 | 0.00 | 0.83 | 0.00 | 4.00 | 2.83 | 0.00 | 18.33 |
| North | Shallow | 2 | Exposed | Fringing | Reserve | 0.00 | 3.36 | 0.00 | 0.00 | 4.92 | 1.12 | 0.00 | 0.00 | 0.00 | 1.44 | 0.00 | 0.52 | 2.40 |
| North | Shallow | 5 | Exposed | Channel | Unprotected | 0.17 | 1.00 | 0.83 | 0.00 | 0.00 | 0.00 | 0.00 | 0.00 | 3.33 | 0.00 | 0.00 | 0.00 | 15.17 |
| South | Deep | 11 | Exposed | Patch | Park | 13.25 | 2.25 | 1.00 | 0.00 | 0.00 | 0.00 | 0.00 | 0.00 | 0.25 | 12.50 | 0.75 | 0.25 | 18.50 |
| North | Shallow | 3 | Sheltered | Channel | Reserve | 0.80 | 0.00 | 0.96 | 1.44 | 0.00 | 0.00 | 0.32 | 0.00 | 0.16 | 0.00 | 5.60 | 2.40 | 16.64 |
| North | Shallow | 3 | Exposed | Patch | Reserve | 0.16 | 1.76 | 0.64 | 0.00 | 0.00 | 0.80 | 0.96 | 0.00 | 0.00 | 0.96 | 0.00 | 0.96 | 8.00 |
| North | Shallow | 3 | Sheltered | Lagoon | Reserve | 1.76 | 3.04 | 0.80 | 0.64 | 0.00 | 5.44 | 0.00 | 1.12 | 0.00 | 0.32 | 1.12 | 0.00 | 10.56 |
| South | Shallow | 4 | Sheltered | Channel | Unprotected | 0.50 | 0.00 | 0.00 | 0.00 | 0.00 | 0.00 | 0.00 | 0.00 | 0.00 | 8.67 | 0.00 | 0.00 | 9.67 |
| North | Shallow | 4.5 | Exposed | Fringing | Reserve | 0.80 | 12.00 | 3.08 | 0.00 | 0.48 | 3.04 | 4.32 | 0.00 | 0.00 | 4.96 | 1.04 | 2.40 | 9.64 |
| Central | Deep | 12 | Exposed | Fringing | Unprotected | 3.33 | 0.00 | 0.33 | 0.00 | 0.00 | 0.00 | 0.00 | 0.00 | 0.00 | 0.00 | 0.00 | 0.00 | 0.33 |
| Central | Shallow | 1 | Sheltered | Patch | Park | 2.33 | 0.00 | 1.17 | 0.50 | 1.67 | 5.33 | 3.33 | 5.83 | 0.00 | 0.33 | 1.17 | 1.83 | 15.17 |
| Central | Shallow | 3 | Exposed | Patch | Park | 0.04 | 0.46 | 0.00 | 0.00 | 0.00 | 0.17 | 0.00 | 0.08 | 0.00 | 0.21 | 0.04 | 0.04 | 0.96 |
| Central | Shallow | 1 | Sheltered | Patch | Park | 1.83 | 5.83 | 0.00 | 0.00 | 0.00 | 0.33 | 1.50 | 1.33 | 0.00 | 0.00 | 5.00 | 2.50 | 9.50 |
| North | Shallow | 6 | Exposed | Fringing | Unprotected | 1.00 | 8.00 | 0.00 | 0.00 | 0.00 | 0.33 | 0.00 | 0.00 | 0.00 | 0.00 | 0.00 | 0.17 | 1.50 |
| Central | Shallow | 1.8 | Sheltered | Lagoon | Park | 4.33 | 5.33 | 2.67 | 0.67 | 2.67 | 0.00 | 0.00 | 0.00 | 0.00 | 0.67 | 0.67 | 0.00 | 2.00 |
| North | Shallow | 2 | Sheltered | Fringing | Reserve | 0.00 | 0.00 | 0.64 | 0.32 | 0.96 | 3.68 | 0.00 | 0.32 | 0.00 | 0.00 | 0.00 | 0.16 | 4.48 |
| North | Shallow | 5 | Exposed | Fringing | Reserve | 0.17 | 2.00 | 2.00 | 0.00 | 0.00 | 2.67 | 0.50 | 3.17 | 0.00 | 4.50 | 0.00 | 0.83 | 12.17 |
| Central | Deep | 7 | Exposed | Fringing | Park | 0.67 | 0.00 | 0.33 | 0.00 | 0.00 | 0.00 | 0.00 | 0.00 | 0.00 | 0.00 | 1.33 | 3.00 | 0.67 |
| South | Deep | 6.5 | Sheltered | Fringing | Reserve | 5.33 | 0.33 | 0.67 | 0.00 | 0.00 | 0.00 | 0.00 | 0.00 | 0.00 | 0.00 | 0.33 | 4.00 | 6.33 |
| South | Deep | 7 | Exposed | Fringing | Reserve | 10.00 | 0.00 | 0.00 | 0.67 | 0.00 | 0.00 | 1.00 | 0.00 | 0.00 | 0.00 | 0.00 | 0.00 | 4.67 |
| Central | Shallow | 1.8 | Sheltered | Lagoon | Park | 4.00 | 0.33 | 0.33 | 2.00 | 0.00 | 0.00 | 0.00 | 0.00 | 0.00 | 0.00 | 6.33 | 2.33 | 4.33 |
