# Supplementary material for: Spatial heterogeneity of coral reef benthic communities in Kenya
Source: PLoS One. 2020 Aug 26;15(8):e0237397. doi: 10.1371/journal.pone.0237397 (PMC7449394; doi:10.1371/journal.pone.0237397)
Supplement: S4 Table — Density (no. of colonies per 100m2) of coral colonies recorded at all surveyed coral reef sites along the Kenyan coast. (DOCX) [file pone.0237397.s004.docx]

| Site name | Geographical zone | Depth category | Depth-m | Exposure | Reef type | Management | Density (no. of colonies per 100m2) | | | | | | | | |
| --- | --- | --- | --- | --- | --- | --- | --- | --- | --- | --- | --- | --- | --- | --- | --- |
|  |  |  |  |  |  |  | 1-2.5cm | 2.6-5cm | 6-10cm | 11-20cm | 21-40cm | 41-80cm | 81-160cm | 161-320cm | >320 |
| Anthias | Central | Deep | 16 | Exposed | Fringing | Unprotected | 25 | 192 | 333 | 155 | 65 | 13 | 3 | 0 | 2 |
| Boso | North | Shallow | 4 | Sheltered | Lagoon | Reserve | 0 | 42 | 108 | 38 | 8 | 2 | 0 | 0 | 0 |
| Chole | North | Shallow | 2 | Exposed | Lagoon | Reserve | 92 | 183 | 250 | 318 | 250 | 114 | 110 | 28 | 26 |
| Chongo cha Bomani | North | Deep | 18 | Exposed | Patch | Reserve | 0 | 8 | 50 | 100 | 56 | 30 | 30 | 8 | 0 |
| Chongo cha Chano | North | Deep | 18 | Exposed | Patch | Reserve | 8 | 8 | 75 | 38 | 6 | 0 | 2 | 0 | 0 |
| Chongo cha Mvundeni | North | Deep | 15 | Exposed | Patch | Reserve | 42 | 150 | 242 | 130 | 38 | 2 | 2 | 0 | 0 |
| Chongo cha Mwongo Shariff | North | Deep | 18 | Exposed | Patch | Reserve | 42 | 25 | 83 | 180 | 56 | 10 | 0 | 0 | 0 |
| Chongo cha Rubu | North | Deep | 18 | Exposed | Patch | Reserve | 58 | 92 | 200 | 24 | 22 | 0 | 0 | 0 | 0 |
| Dolphin | Central | Deep | 13 | Exposed | Fringing | Unprotected | 125 | 175 | 358 | 152 | 45 | 13 | 0 | 0 | 0 |
| Drummers | Central | Deep | 13 | Exposed | Fringing | Unprotected | 100 | 225 | 125 | 138 | 58 | 23 | 2 | 0 | 0 |
| Fawacho | North | Shallow | 5 | Sheltered | Channel | Unprotected | 72 | 89 | 17 | 61 | 36 | 16 | 12 | 8 | 8 |
| Kibuyuni | South | Shallow | 2 | Sheltered | Channel | Unprotected | 42 | 100 | 175 | 190 | 178 | 72 | 30 | 28 | 8 |
| Kishanga | North | Shallow | 3 | Sheltered | Lagoon | Reserve | 0 | 25 | 75 | 84 | 82 | 16 | 12 | 2 | 0 |
| Kisite Deep | South | Deep | 10 | Sheltered | Fringing | Park | 58 | 158 | 200 | 165 | 163 | 45 | 7 | 0 | 0 |
| Kisite Leeward | South | Shallow | 5 | Sheltered | Fringing | Park | 31 | 88 | 38 | 68 | 56 | 25 | 9 | 1 | 4 |
| Kisite Seaward | South | Shallow | 6 | Exposed | Fringing | Park | 150 | 338 | 513 | 374 | 191 | 76 | 14 | 0 | 1 |
| Kui | North | Shallow | 5 | Sheltered | Lagoon | Reserve | 50 | 92 | 142 | 88 | 40 | 56 | 24 | 30 | 26 |
| Kupi | North | Shallow | 4 | Sheltered | Lagoon | Reserve | 158 | 175 | 258 | 178 | 74 | 62 | 34 | 0 | 8 |
| Kwa Radi | North | Shallow | 2 | Exposed | Fringing | Reserve | 0 | 33 | 17 | 76 | 68 | 42 | 10 | 0 | 0 |
| Mabiyu | North | Shallow | 5 | Exposed | Channel | Unprotected | 200 | 117 | 250 | 112 | 60 | 50 | 20 | 20 | 6 |
| Makokokwe | South | Deep | 11 | Exposed | Patch | Park | 25 | 63 | 181 | 128 | 146 | 129 | 36 | 1 | 5 |
| Mikes Inner | North | Shallow | 3 | Sheltered | Channel | Reserve | 8 | 133 | 50 | 122 | 128 | 216 | 118 | 24 | 20 |
| Mikes Outer | North | Shallow | 3 | Exposed | Patch | Reserve | 25 | 108 | 92 | 70 | 76 | 70 | 54 | 14 | 24 |
| Mkokoni | North | Shallow | 3 | Sheltered | Lagoon | Reserve | 17 | 25 | 67 | 10 | 32 | 22 | 12 | 34 | 24 |
| Mkwiro | South | Shallow | 4 | Sheltered | Channel | Unprotected | 158 | 250 | 325 | 242 | 62 | 13 | 7 | 7 | 2 |
| Mlango wa Muhindi | North | Shallow | 4.5 | Exposed | Fringing | Reserve | 92 | 158 | 300 | 150 | 122 | 58 | 16 | 6 | 12 |
| Moray | Central | Deep | 12 | Exposed | Fringing | Unprotected | 175 | 292 | 250 | 112 | 35 | 13 | 3 | 0 | 2 |
| New Coral Gardens | Central | Shallow | 1 | Sheltered | Patch | Park | 133 | 150 | 508 | 112 | 66 | 58 | 26 | 12 | 4 |
| North Reef | Central | Shallow | 3 | Exposed | Patch | Park | 167 | 192 | 325 | 90 | 56 | 44 | 22 | 8 | 4 |
| Old Coral Gardens | Central | Shallow | 1 | Sheltered | Patch | Park | 175 | 108 | 183 | 92 | 58 | 46 | 28 | 4 | 4 |
| Pezzali | North | Shallow | 6 | Exposed | Fringing | Unprotected | 300 | 283 | 550 | 192 | 78 | 16 | 10 | 10 | 4 |
| Richard Bennette | Central | Shallow | 1.8 | Sheltered | Lagoon | Park | 133 | 100 | 25 | 50 | 74 | 58 | 10 | 22 | 26 |
| Shili | North | Shallow | 2 | Sheltered | Fringing | Reserve | 17 | 58 | 17 | 8 | 20 | 10 | 4 | 0 | 0 |
| Shimo La Tewa | North | Shallow | 5 | Exposed | Fringing | Reserve | 42 | 83 | 100 | 194 | 92 | 74 | 72 | 20 | 14 |
| Turtle Reef | Central | Deep | 7 | Exposed | Fringing | Park | 0 | 58 | 0 | 78 | 62 | 42 | 6 | 4 | 4 |
| Upper Mpunguti Leeward | South | Deep | 6.5 | Sheltered | Fringing | Reserve | 150 | 67 | 225 | 215 | 192 | 37 | 7 | 3 | 0 |
| Upper Mpunguti Seaward | South | Deep | 7 | Exposed | Fringing | Reserve | 200 | 233 | 292 | 298 | 182 | 32 | 5 | 0 | 0 |
| Watamu Coral Garden | Central | Shallow | 1.8 | Sheltered | Lagoon | Park | 75 | 58 | 175 | 46 | 64 | 36 | 8 | 2 | 6 |
