# Supplementary material for: Spatial heterogeneity of coral reef benthic communities in Kenya
Source: PLoS One. 2020 Aug 26;15(8):e0237397. doi: 10.1371/journal.pone.0237397 (PMC7449394; doi:10.1371/journal.pone.0237397)
Supplement: S5 Table — Summary of all studied sites along the Kenyan coast. (DOCX) [file pone.0237397.s005.docx]

| Major benthic category |  | % Mean cover | sd |
| --- | --- | --- | --- |
| Biotic benthic communities | Hard coral | 24.12 | 26.72 |
|  | Crustose Coralline algae | 6.74 | 12.83 |
|  | Halimeda | 6.57 | 13.01 |
|  | Macroalgae | 12.88 | 22.74 |
|  | Turf algae | 16.21 | 21.77 |
|  | Dead standing coral | 0.56 | 2.66 |
|  | Soft coral | 7.00 | 16.13 |
|  | Seagrass | 1.07 | 6.41 |
|  | Other-Invertebrates | 1.61 | 5.52 |
|  | Recently dead coral | 0.24 | 1.51 |
| Abiotic benthic categories | Bare substrate | 10.93 | 16.98 |
|  | Rubble | 8.26 | 18.56 |
|  | Sand | 1.44 | 5.50 |
|  | Silt | 1.88 | 9.03 |
|  | Other-unidentified | 0.49 | 2.04 |
