# Supplementary material for: Spatial heterogeneity of coral reef benthic communities in Kenya
Source: PLoS One. 2020 Aug 26;15(8):e0237397. doi: 10.1371/journal.pone.0237397 (PMC7449394; doi:10.1371/journal.pone.0237397)
Supplement: S6 Table — Summary of all studied sites along the Kenyan coast. (DOCX) [file pone.0237397.s006.docx]

| Coral genera | % Mean cover | sd |
| --- | --- | --- |
| Porites | 7.66 | 17.61 |
| Acropora | 2.12 | 7.26 |
| Echinopora | 2.06 | 10.07 |
| Montipora | 1.64 | 7.99 |
| Goniopora | 1.02 | 8.56 |
| Favites | 1.00 | 5.32 |
| Platygyra | 0.88 | 4.86 |
| Goniastrea | 0.73 | 6.50 |
| Pocillopora | 0.64 | 3.08 |
| Galaxea | 0.59 | 5.54 |
| Gardineroseris | 0.55 | 5.28 |
| Leptoria | 0.53 | 4.93 |
| Lobophyllia | 0.47 | 4.88 |
| Hydnophora | 0.44 | 4.32 |
| Pavona | 0.43 | 4.44 |
| Turbinaria | 0.38 | 4.56 |
| Echinophyllia | 0.34 | 3.59 |
| Dipsastrea | 0.34 | 2.24 |
| Fungia | 0.33 | 2.98 |
| Alveopora | 0.29 | 3.12 |
| Coscinaraea | 0.24 | 2.63 |
| Astreopora | 0.24 | 2.43 |
| Acanthastrea | 0.16 | 2.45 |
| Cyphastrea | 0.11 | 1.46 |
| Seriatopora | 0.11 | 1.17 |
| Paramontastrea | 0.11 | 2.19 |
| Tubastrea | 0.10 | 2.18 |
| Millepora | 0.06 | 1.58 |
| Isopora | 0.06 | 1.32 |
| Psammocora | 0.05 | 1.16 |
| Heliopora | 0.04 | 0.72 |
| Leptoseris | 0.03 | 0.86 |
| Pachyseris | 0.03 | 0.86 |
| Stylophora | 0.02 | 0.35 |
| Herpolitha | 0.01 | 0.14 |
| Leptastrea | 0.01 | 0.14 |
| Diploastrea | 0.00 | 0.00 |
| Merulina | 0.00 | 0.00 |
| Physogyra | 0.00 | 0.00 |
| Plerogyra | 0.00 | 0.00 |
| Plesiastrea | 0.00 | 0.00 |
| Siderastrea | 0.00 | 0.00 |
| Tubipora | 0.00 | 0.00 |
