# Supplementary material for: Spatial heterogeneity of coral reef benthic communities in Kenya
Source: PLoS One. 2020 Aug 26;15(8):e0237397. doi: 10.1371/journal.pone.0237397 (PMC7449394; doi:10.1371/journal.pone.0237397)
Supplement: S7 Table — Summary of all studied sites along the Kenyan coast. (DOCX) [file pone.0237397.s007.docx]

| Coral size-class | Mean density  (no. of colonies per 100m^2^) | sd |
| --- | --- | --- |
| 1-2.5cm | 85.35 | 93.48 |
| 2.6-5cm | 133.24 | 114.67 |
| 6-10cm | 197.07 | 182.12 |
| 11-20cm | 136.58 | 108.21 |
| 21-40cm | 85.93 | 72.79 |
| 41-80cm | 43.41 | 45.80 |
| 81-160cm | 18.58 | 28.25 |
| 161-320cm | 6.77 | 12.40 |
| >320cm | 5.70 | 10.36 |
