# Supplementary material for: Spatial heterogeneity of coral reef benthic communities in Kenya
Source: PLoS One. 2020 Aug 26;15(8):e0237397. doi: 10.1371/journal.pone.0237397 (PMC7449394; doi:10.1371/journal.pone.0237397)
Supplement: S8 Table — (DOCX) [file pone.0237397.s008.docx]

| **Benthic**  **community** | **Source** | **df** | **SS** | **MS** | **Pseudo-F** | **P(perm)** | **Unique**  **perms** |
| --- | --- | --- | --- | --- | --- | --- | --- |
| Benthic cover | Ge | 2 | 5757 | 2878 | 4.140 | 0.001 | 999 |
|  | Res | 35 | 24336 | 695 |  |  |  |
|  | Total | 37 | 30093 |  |  |  |  |
| Coral genera | Ge | 2 | 8358 | 4179 | 2.628 | 0.004 | 998 |
|  | Res | 35 | 55659 | 1590 |  |  |  |
|  | Total | 37 | 64016 |  |  |  |  |
| Coral size class | Ge | 2 | 2091 | 1046 | 2.3595 | 0.033 | 998 |
|  | Res | 35 | 15510 | 443 |  |  |  |
|  | Total | 37 | 17602 |  |  |  |  |
