# Supplementary figures and images for: Spatial heterogeneity of coral reef benthic communities in Kenya
Source: PLoS One. 2020 Aug 26;15(8):e0237397. doi: 10.1371/journal.pone.0237397 (PMC7449394; doi:10.1371/journal.pone.0237397)

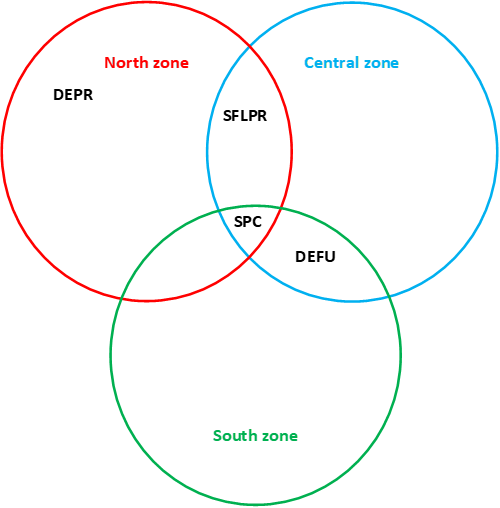

Supplement: S1 Fig — North zone has three habitat types one of them uniquely to this zone (DEPR); Central zone has three habitat types, one it shares with all other zones (SPC), another with only north (SFLPR) and only south zone (DEFU); South zone has only two habitat types, one it shares with all zones (SCP) and the other with only Central zone (DEFU). (TIF) [file pone.0237397.s009.tif]
